# Supplementary material for: Clozapine-N-oxide impairs spatial memory independent of DREADDs
Source: Front Cell Neurosci. 2026 May 8;20:1785079. doi: 10.3389/fncel.2026.1785079 (PMC13194068; doi:10.3389/fncel.2026.1785079)
Supplement: Supplementary file 1 [file Table_1.DOCX]

**Supplementary Table 1. Training discrimination index (DI) by treatment group.** Group differences were analyzed using one-way ANOVA, which showed no significant effect of treatment, *F*(2, 31) = 3.014, *p* = 0.0636.

| **Treatment group** | **n** | **Mean ± SEM** | **SD** |
| --- | --- | --- | --- |
| Vehicle | 17 | 0.072 ± 0.023 | 0.09 |
| CNO | 8 | 0.065 ± 0.034 | 0.09 |
| C21 | 9 | -0.013 ± 0.020 | 0.06 |

**Supplementary Table 2. Total exploration time during OLM training by treatment group.** Group differences were analyzed using one-way ANOVA, which showed no significant effect of treatment, *F*(2, 31) = 1.20, *p* = 0.3137.

| **Treatment group** | **n** | **Mean ± SEM (s)** | **SD (s)** |
| --- | --- | --- | --- |
| Vehicle | 17 | 47.6 ± 5.26 | 21.7 |
| CNO | 8 | 55.9 ± 5.97 | 16.9 |
| C21 | 9 | 41.4 ± 5.29 | 15.9 |

**Supplementary Table 3. Total exploration time during OLM test by treatment group.** Group differences were analyzed using the Kruskal–Wallis test, which showed no significant effect of treatment, Kruskal–Wallis statistic = 5.54, *p* = 0.0628.

| **Treatment group** | **n** | **Mean ± SEM (s)** | **SD (s)** |
| --- | --- | --- | --- |
| Vehicle | 17 | 29.1 ± 2.53 | 10.4 |
| CNO | 8 | 36.4 ± 4.98 | 14.1 |
| C21 | 9 | 24.8 ± 3.27 | 9.80 |

**Supplementary Table 4. Total distance moved during OLM training by treatment group.** Group differences were analyzed using the Kruskal–Wallis test, which showed no significant effect of treatment, Kruskal–Wallis statistic = 2.24, *p* = 0.3266.

| **Treatment group** | **n** | **Mean ± SEM (cm)** | **SD (cm)** |
| --- | --- | --- | --- |
| Vehicle | 17 | 2340 ± 81.0 | 334 |
| CNO | 8 | 2479 ± 63.0 | 178 |
| C21 | 9 | 2053 ± 234 | 703 |

**Supplementary Table 5. Total distance moved during OLM test by treatment group.** Group differences were analyzed using one-way ANOVA, which showed no significant effect of treatment, *F*(2, 31) = 0.288, *p* = 0.7520.

| **Treatment group** | **n** | **Mean ± SEM (cm)** | **SD (cm)** |
| --- | --- | --- | --- |
| Vehicle | 17 | 1985 ± 87.7 | 361 |
| CNO | 8 | 1944 ± 71.1 | 201 |
| C21 | 9 | 2056 ± 95.2 | 286 |
